# Novel *THPO* variant in hereditary thrombocytopenia: A potential candidate variant for predisposition to myeloid neoplasm

**DOI:** 10.1371/journal.pone.0271624

**Published:** 2022-12-19

**Authors:** Seok Ryun Kwon, Man Jin Kim, Young-eun Lee, Jiwon Yun, Da-jeong Jeong, Jae Hyeon Park, Sunghoon Kwon, Dong Soon Lee

**Affiliations:** 1 Department of Laboratory Medicine, Seoul National University College of Medicine, Seoul, Korea; 2 Department of Genomic Medicine, Seoul National University Hospital, Seoul, Korea; 3 Department of Electrical and Computer Engineering, Seoul National University, Seoul, Korea; 4 Cancer Research Institute, Seoul National University College of Medicine, Seoul, Korea; Qatar University, QATAR

## Abstract

Hereditary thrombocytopenia is a heterogeneous group of congenital disorders with a wide range of symptoms depending on the severity of platelet dysfunction or thrombocytopenia. Because of its clinical phenotypes and the bone marrow morphology associated with this condition, hereditary thrombocytopenia can be misdiagnosed as primary immune thrombocytopenia and myelodysplastic syndrome. Therefore, genetic evidence is necessary for the accurate diagnosis of hereditary thrombocytopenia. Refractory cytopenia of childhood is a subgroup of myelodysplastic syndrome that was added to the World Health Organization classification in 2008. To investigate the germline and somatic variants associated with refractory cytopenia of childhood, we performed targeted multigene sequencing in three patients with refractory cytopenia of childhood. Of the three patients, one progressed from megakaryocytic hypoplasia with thrombocytopenia, and targeted multigene sequencing revealed *THPO* variants in this patient and his sister. We propose that the monoallelic deletion of *THPO* is a potential candidate for germline predisposition to myeloid malignancy.

## Introduction

Hereditary thrombocytopenia is a heterogeneous group of congenital disorders with a wide spectrum of symptoms influenced by the severity of platelet dysfunction or thrombocytopenia [1]. Many patients with hereditary thrombocytopenia show mild or no bleeding symptoms, and some develop thrombocytopenia late in adulthood [2]. Unlike inherited bone marrow failure syndrome, hereditary thrombocytopenia does not accompany the underlying disease; thus, it can go underdiagnosed. Moreover, it is misdiagnosed as primary immune thrombocytopenia in a significant proportion of patients [3, 4]. Furthermore, the bone marrow histology features observed in hereditary thrombocytopenia can occasionally be similar to those observed in myelodysplastic syndrome with single lineage dysplasia [5], and hereditary thrombocytopenia may be misdiagnosed as myelodysplastic syndrome (MDS) [6]. Consequently, hereditary thrombocytopenia patients without an accurate diagnosis are at risk of incorrect treatment, such as splenectomy or chemotherapy. Therefore, strong genetic evidence is important for the accurate diagnosis of hereditary thrombocytopenia [7].

The process of platelet formation involves megakaryopoiesis, platelet production, and platelet clearance. Each process involves several pathways controlled by the expression of specific genes and functions as a feedback loop. Genetic mutations during thrombopoiesis can result in inherited platelet disorders. Depending on the genetic alterations at each stage, the phenotypes of hereditary thrombocytopenia show a broad spectrum with variable platelet numbers, sizes, and dysfunctions. The thrombopoietin-encoding *THPO* (thrombopoietin) gene is a growth factor essential for hematopoietic stem cell survival. The megakaryocyte maturation and thrombopoietin receptor encoding gene is *MPL*. Gene variants of *THPO* and *MPL* cause *THPO*-related thrombocytopenia and congenital amegakaryocytic thrombocytopenia (CAMT), respectively. To date, at least 40 genes have been reported in hereditary thrombocytopenia. Among these gene-related disorders, familial platelet disorders with associated myeloid malignancy (FPD/AML), ankyrin repeat domain containing 26 related thrombocytopenia (ANKRD26-RT), and ETS variant transcription factor 6-related thrombocytopenia (ETV6-RT) require mutational screening of potential hematopoietic stem cell transplantation sibling donors, as patients with these gene variants have a high risk of developing hematological malignancies [8, 9]. The proposed guidelines on the examination of inherited thrombocytopenia recommend that genotyping of *THPO* is necessary for the diagnosis of *THPO*-immune thrombocytopenia [1].

Refractory cytopenia of childhood (RCC) is a subgroup of MDS and was added to the World Health Organization classification of tumors of hematopoietic and lymphoid tissues (WHO criteria) in 2008. RCC was formerly classified as childhood aplastic anemia [10, 11]. RCC, when presented as thrombocytopenia, is often confused with immune thrombocytopenia or inherited thrombocytopenia. Currently, known germline variants associated with RCC syndrome are *VHL* (von Hippel–Lindau syndrome), *MET* (hereditary papillary RCC), *FH* (hereditary leiomyomatosis and RCC), *TSC1/2* (tuberous sclerosis complex), *FLCN* (Birt-Hogg-Dubé syndrome), *SDHA/B/C/D* (hereditary pheochromocytoma and paraganglioma), *PTEN* (Cowden syndrome) and *BAP1* (BAP1 tumor predisposition syndrome) genes. The non-RCC genes include *TPS3*, *ATM*, *BRCA1*, *CHEK2*, *RET*, *PALB2*, *BARD1*, *APC*, *MUTYH*, *FANCC*, *RADSO*, *RECQL*, and *RECQL4*.

To investigate the germline and somatic variants of RCC, we performed multitarget gene sequencing in three patients with RCC, of whom one child progressed from megakaryocytic hypoplasia with thrombocytopenia and was further studied. Targeted multigene sequencing revealed *THPO* variants in the proband and his sister. Here, we propose a monoallelic deletion in *THPO* as a possible candidate variant of germline predisposition to myeloid malignancies.

## Materials and methods

### Patients and ethics statement

Three patients diagnosed with RCC between 2010, and 2016 at Seoul National University Hospital were enrolled in this study. The diagnosis was based on the WHO criteria [12]. Laboratory data, including age, sex, complete blood count, bone marrow morphology, diagnosis, and therapy, were obtained for each patient. Of the three patients, we conducted familial analysis in one child suspected of hereditary thrombocytopenia. Peripheral blood and saliva samples were collected from the patient’s family. This study was approved by the Institutional Review Board of Seoul National University Hospital (IRB No. 1311-091-535), and written informed consent was obtained from all the patients and their family members.

### Bone marrow examination

Bone marrow smears were stained with Wright-Giemsa, and the biopsies (sections) were subjected to hematoxylin and eosin (H&E) and immunohistochemical (IHC) staining. Paraffin-embedded tissue blocks were trimmed and sliced into 2-μm-thick sections. The slides were incubated at 56°C for 30 min and hydrated with xylene, 100% ethanol (EtOH), 95% EtOH, and 70% EtOH. They were then incubated with anti-CD34 monoclonal antibody (Leica Biosystems, Newcastle, UK), anti-CD117 monoclonal antibody (Dako, Copenhagen, Denmark), and anti-CD61 monoclonal antibody (Roche Diagnostics, Mannheim, Germany) for 15 min at room temperature. Subsequently, the slides were dehydrated using 70% EtOH, 95% EtOH, 100% EtOH, and xylene. The proportion of blasts and morphologic dysplasia in the three hematopoietic lineages, namely, myeloids, erythroids and lymphocytes, was examined using bone marrow aspiration smears. In the bone marrow sections, cellularity, blast infiltration, and megakaryocyte enumeration were also evaluated.

### G-banding and fluorescent in situ hybridization (FISH) analysis

Chromosomal analysis was performed using the conventional G-banding technique. Heparinized bone marrow samples were collected, and white blood cells (WBCs) were sorted by centrifugation and cultured in RPMI-1640 medium (Thermo Fisher Scientific) at 37°C in 5% CO_2_ for 24 h. They were then treated with colcemid to inhibit mitosis. The specimen in the medium was centrifuged, and the upper layer was decanted. KCl was added at 37°C for 20 min. For fixation, 1 mL of Carnoy’s solution was used. The slides were then subjected to Leishman’s G-banding staining according to the standard protocol. A minimum of 20 metaphase cells per patient were analyzed using Metafer 4 software (MetaSystems, Altlussheim, FRG). The karyotype designation was based on the principles of the International System for Human Cytogenetic Nomenclature (ISCN 2016).

Interphase FISH analysis was performed on mononuclear bone marrow cell aspirates to detect common cytogenetic abnormalities related to MDS and/or aplastic anemia using LSI EGR, LSI D7S522 (Vysis), LSI 20 (Vysis), LSI CEP 8 (Vysis), and LSI 1q25 (Vysis) probes and those related to acute myeloid leukemia using LSI PML/RARA (Vysis), LSI MLL (Vysis), LSI AML1/ETO (Vysis), LSI CBFB (Vysis), and LSI RPN1/MECOM (Vysis) probes. For FISH slide preparation, each bone marrow aspirate specimen was combined with 10 mL of 0.075 M KCl and was then centrifuged at 1200 rpm for 8 min. The supernatant was separated, and the pellet was incubated with 0.075 M KCl in a 37°C water bath for 30 min. A solution containing methanol and acetic acid in a 3:1 ratio was used for fixation. The slides were immersed in 0.1% NP40/2× sodium saline citrate (SSC) for 30 min at 37°C and dehydrated with 70%, 85%, and 100% EtOH for 3 min each. The slides were then air-dried. The probes were prepared in the dark by mixing 7 μL of hybridization buffer, 1 μL of LSI probe, and deionized water. A total of 10 μL of the probe mixture was dropped on the FISH slides. The slides and probes were then denatured at 75°C for 3 min. They were then left overnight for hybridization at 39°C, following which they were prewarmed in a solution containing 0.3% nonylphenol polyethylene glycol (NP-40) and 0.4% SSC at 73°C for 2 min. Then, 6.6 μL of 4′,6-diamidino-2-phenylindole (DAPI II) (Vysis) was dropped on each slide for counterstaining. Fluorescent signals were visualized using a fluorescence microscope (Zeiss, Germany). At least 200 cells were analyzed for each specimen. FISH results were recorded according to the ISCN 2016 guidelines. The nuclei with ambiguous signals and cells with poor morphology were excluded from scoring. To set the normal reference range for each FISH probe, control experiments were performed on bone marrow samples from patients free of hematologic diseases (uncultured mononuclear cells from the peripheral blood of 40 healthy persons free of hematologic malignancies). The mean ± 3 standard deviations (SDs) of the normal range were used as the reference range.

### Targeted multigene sequencing

We selected 647 genes associated with hematologic disorders, including inherited platelet disorders [1, 2] and myeloid malignancy [13–15], for targeted sequencing by next-generation sequencing (NGS) (S1 Table). To construct a sequencing library, genomic DNA (gDNA) was extracted from the buffy coat of bone marrow aspirates sampled in anticoagulated with sodium ethylenediamine tetraacetate. After lysis of red blood cells and washing, cell pellets were resuspended in RNAlater solution and stored at -80°C. Total gDNA extraction was performed using the QIAamp DNA Blood Mini Kit (Qiagen, Valencia, CA, USA) following the manufacturer’s instructions. gDNA shearing, standard library preparation, and hybridization were performed by Celemics Inc. (Seoul, Korea). Sheared gDNA quality was evaluated using an Agilent 2200 TapeStation System (Agilent, Santa Clara, CA, USA). A target template length of 259 kb was sequenced at the paired-end 150 bp rapid-run sequencing mode on an Illumina HiSeq 2500 platform (Illumina, San Diego, CA, USA) according to the manufacturer’s instructions.

### Variant calling strategy

The Fastq files obtained after targeted sequencing were aligned to the human reference sequence (hg19, GRCh37) using the Burrows–Wheeler Aligner (BWA, v0.7). The potential PCR duplicates were removed using Picard MarkDuplicates (http://broadinstitute.github.io/picard), and reliable variants were determined using Unified Genotyper in GATK 4.2 using GATK HaplotypeCaller software. To filter out low-quality variants, those data with a total depth below 20 and a low altered allele count (<10) were discarded. Synonymous single-nucleotide variants and noncoding variants were also filtered out. Subsequently, variants with more than 0.01 allele frequency based on dbSNP137, dbSNP138, ExAC, EVS(ESP6500), and 1000 Genomes were filtered out. In addition, an in-house Korean single nucleotide polymorphism database (KRGDB) was used to filter out common variants in the normal Korean population. Annotation of the variants was performed using ANNOVAR software. All filtered variants were manually verified using Integrative Genomics Viewer (IGV) and AlamutVisual-2.15. The functional effects of missense variants were predicted using the in silico tools SIFT, PolyPhen-2 HDIV, MutationTaster, MutationAssessor, FATHMM, and CADD.

### Sanger sequencing of the *THPO* variant

Sanger sequencing was performed to verify the deleterious variant in *THPO* (NM_001290003.1: c.388_389delAG, p. Arg130Glyfs*15) observed in the familial analysis of patient 1. The following primers were designed for the *THPO* variant: forward primer: 5′-AGGCTGGTCAGCATCTCAAG-3′, and reverse primer: 5′-CACAATACAGCCCGCATTTA-3′.

## Results

### Demographics of three patients with MDS

The mean age of the three patients with RCC was 16 years (range, 9–24) (Table 1). All patients showed thrombocytopenia and bleeding tendency. The median values of the hemogram were 8.5 g/dL for the hemoglobin level (8.2–8.9), 2.41 (×10^9^/L) for the WBC count (1.31–4.10), and 29 (×10^9^/L) for the platelet level (5–55). All patients had progressed to leukemic transformation and denied a family history of hematologic malignancy. Two patients were suspected of having predisposition syndrome, which is the CAMT, of MDS (Table 2).

**Table 1:** Clinical characteristics and laboratory findings of three patients with refractory cytopenia of childhood.

Characteristics	Total (N = 3)
Age*	16 (9–24)
Sex	
Male	3
RCC, subtype	
MDS-EB2	3
Dysplastic lineages	
Unilineage	2
Multilineage	1
Cytogenetics	
MDS defining abnormality	1
Any others	1
Symptoms	
Thrombocytopenia	3
Bleeding tendency	3
Leukemic transformation	3
Hematologic parameters*	
Hb (g/l)	8.5 (8.2–8.9)
WBC (×10^9^/l)	2.41 (1.31–4.10)
Platelet (×10^9^/l)	29 (5–55)
BM blast (%)	16.5 (11.4–19.2)
PB blast (%)	4 (0–10)

RCC, refractory cytopenia of childhood; MDS, myelodysplastic syndrome; WBC, white blood cell; BM, bone marrow; PB, peripheral blood.

*Values are presented as medians (ranges).

**Table 2:** Clinical information and bone marrow findings of G-banding/FISH and targeted multigene sequencing in the three patients with myelodysplastic syndrome.

No		Primary diagnosis	Progressive duration	BM diagnosis	Predisposition syndromes	G-banding	Multigene sequencing	Reference sequence
						FISH		
1	M/9	MDS/MPN-U	1 y	MDS EB2	CAMT	*46,XY[20]	*THPO*, c.388_389delAG, p.Arg130Glyfs*15	NM_001290003
						†nuc ish(D5S23,EGR1)×2[200] nuc ish(D7Z1,D7S522)×2[200] nuc ish(D8Z2×2)[200] nuc ish(BAK1,MAPRE1,BPIL1.PTPRT)×2[200] nuc ish(CEB108,P58,ABL2,ANGPTL)×2[200]	*NHP2*, c.70T>A, p.Tyr24Asn	NM_017838
2	M/16	MDS EB2	8 m	MDS EB2 Erythroid predominance	N/A	47,XY,+8[17]/46,XY[3]	*DDX41*, c.1165G>C, p.Ala389Pro	NM_016222
						nuc ish(D8Z2×3)[90/200] nuc ish(ETO×3,AML1×2)[88/200] nuc ish(D5S23,EGR1)×2[200] nuc ish(D7Z1,D7S522)×2[200] nuc ish(CEB108,P58,ABL2,ANGPTL)×2[200] nuc ish(PTPRT,MYBL2)×2[200] nuc ish(CBFB×2)[200] nuc ish(PML,RARA)×2[200] nuc ish(MLL×2)[200]		
3	M/24	Multiple MDS	21 y	MDS EB2	CAMT	46,XY,t(3;21)(q26;q22)[18]/46,XY[2]	*BCOR*, c.4071+1G>A, Splicing	NM_017745
						nuc ish(3’MECOM×2,5’MECOM×3)(3’MECOM con 5’MECOM×2)[96/300]	*FANCM*, c.2996C>T, p.Pro999Leu	NM_020937

M: male, MDS: myelodysplastic syndromes, PB: peripheral blood, BM: bone marrow, FISH: fluorescence in situ hybridization, MDS/MPN-U: myelodysplastic syndromes/myeloproliferative neoplasm-unclassifiable, MDS-EB2: myelodysplastic syndromes with excess blasts-2, CAMT: congenital amegakaryocytic thrombocytopenia, y: year, m: month, N/A: not applicable.

*, † G-banding and FISH were not performed at MDS-EB2, the results were at primary diagnosis which was MDS/MPN-U

### Bone marrow features of the three patients with MDS

**Fig 1:**
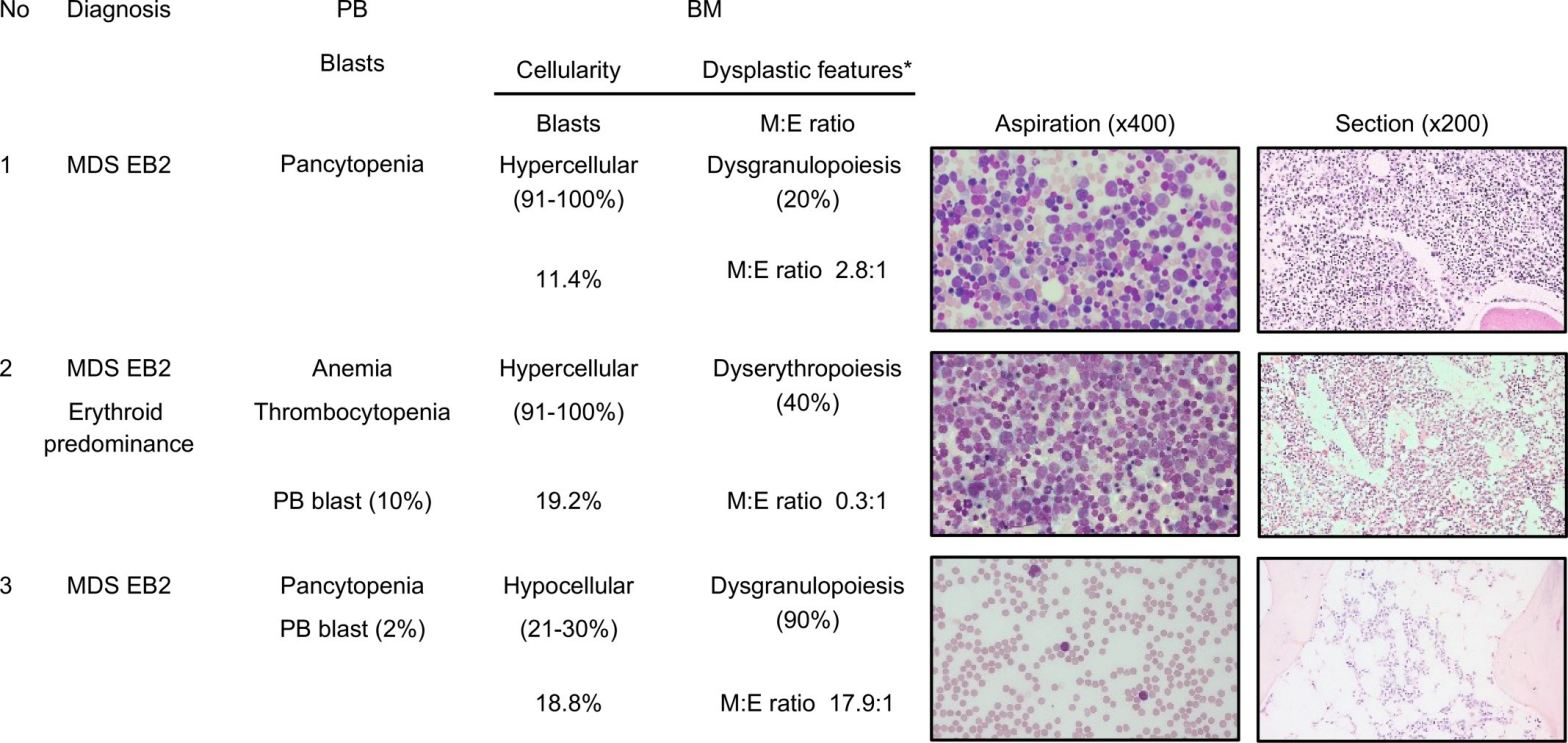
Summary of bone marrow examinations of the three patients with refractory cytopenia of childhood (RCC). BM, bone marrow; MDS, myelodysplastic syndromes; BM, bone marrow; MDS-EB2, myelodysplastic syndromes with excess blasts-2.

The three patients with RCC were diagnosed with myelodysplastic syndrome with excess blasts-2 (MDS-EB2) (Fig 1). Patient 1 exhibited peripheral pancytopenia with dysgranulopoiesis and an increase in the number of blasts (11.4%) on bone marrow aspiration. Patient 2 exhibited anemia and thrombocytopenia with prominent dyserythropoietic features and an increase in the number of blasts (19.2%) on bone marrow aspiration. Patient 3 exhibited pancytopenia with dyserythropoiesis and an increase in the number of blasts (18.8%) on bone marrow aspiration.

### Cytogenetic analysis of the three patients with MDS

The G-banding results are summarized in Table 2. G-banding analysis revealed cytogenetic aberrations in two of the three (66.7%) patients. The aberrations were as follows: 47,XY,+8[17]/46,XY[3] and 46,XY,t(3;21)(q2t6;q22)[18]/46,XY[2]. Trisomy 8 and *MECOM* gene (3q26.2) rearrangements were detected in 33.3% of patients (1/3 patients).

### Targeted multigene sequencing of samples from the three patients with MDS

**Fig 2:**
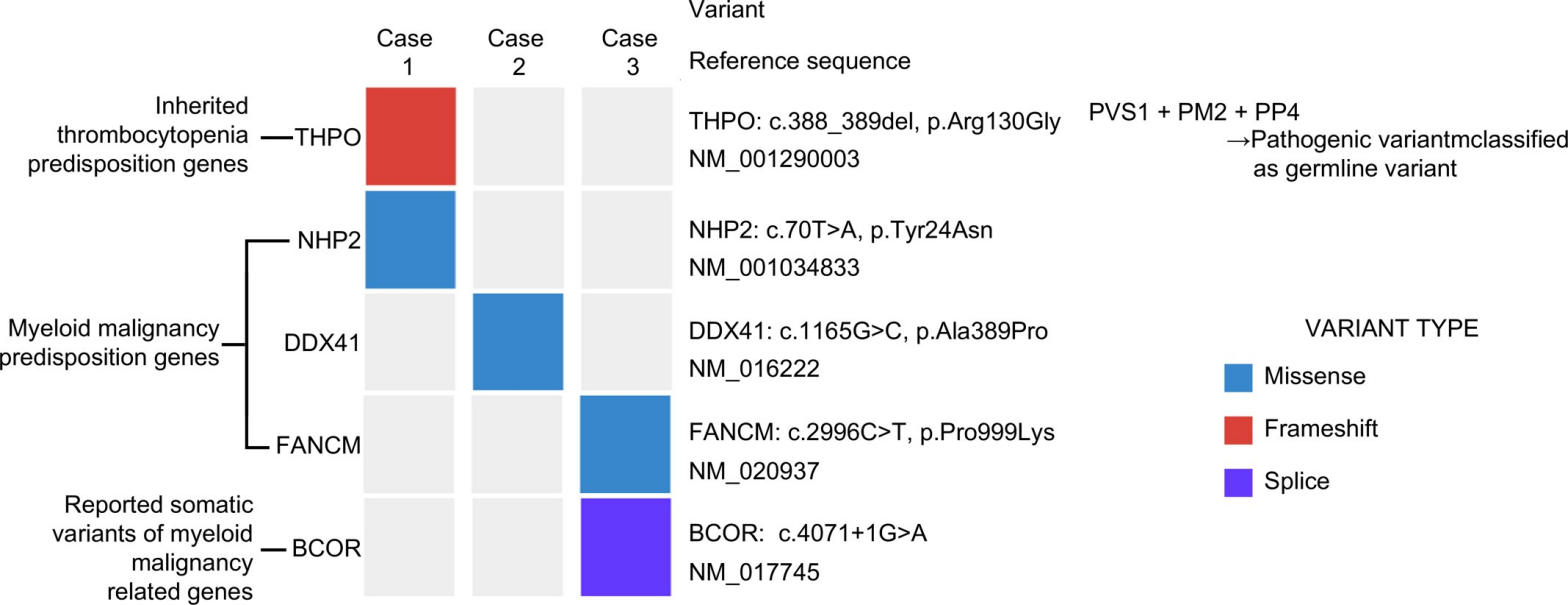
Heatmap showing gene variants of three MDS-EB2 patients with refractory cytopenia of childhood. Of all variants filtered out by targeted multigene sequencing performed on three patients, only those observed in the myeloid malignancy predisposition genes, inherited thrombocytopenia predisposition genes, and somatic variants of myeloid malignancy-related genes are represented. A frameshift variant in *THPO* and a missense variant in *NHP2* were observed in patient 1. A missense variant in *DDX41* was observed in patient 2. A missense variant in *FANCM* and a splicing variant in *BCOR* were observed in patient 3. Inherited thrombocytopenia predisposition genes were identified based on guidelines in Bury et al. [1]. Myeloid malignancy predisposition genes were identified based on the WHO criteria and NCCN guidelines. The somatic variant was identified based on the NCCN guidelines and reported in the COSMIC database. MDS-EB2, myelodysplastic syndromes with excess blasts-2; WHO criteria, World Health Organization classification of tumors of hematopoietic and lymphoid tissues; NCCN, The National Comprehensive Cancer Network; COSMIC, Catalog Of Somatic Mutations In Cancer.

Targeted multigene sequencing was performed on frozen bone marrow specimens obtained from the three patients with MDS (Table 2). Among somatic variants, all filtered variants, including the splice variant of *BCOR*, were classified as Tier 3 variants [16]. Patient 1 harbored a *THPO* variant, which was classified as Tier 3 in the classification of somatic variants. The variant allele frequency of all listed genes was under 0.3–0.7 and 0.8–1.0. However, because of the use of a bone marrow sample, it was difficult to clearly distinguish between germline and somatic variants; therefore, all filtered variants were reclassified according to the American College of Medical Genetics and Genomics (ACMG) guidelines [17]. According to the ACMG guidelines, the germline variant *THPO*:c.388_389delAG, p. Arg30Gly frameshift observed in patient 1 was classified as a pathogenic variant by PVS1 (frameshift), PM2 (east Asian allele frequency 0.011%), and PP4 (thrombocytopenia phenotype for the *THPO* gene), and the other listed gene variants were classified as VUS (variants of uncertain significance). Of all filtered variants in the three MDS-EB2 patients, those in inherited thrombocytopenia predisposition genes, myeloid malignancy predisposition genes, and somatic variants of myeloid malignancy-related genes are shown in Fig 2.

### Clinical history and bone marrow examination of the patient 1 with the *THPO* variant

**Fig 3:**
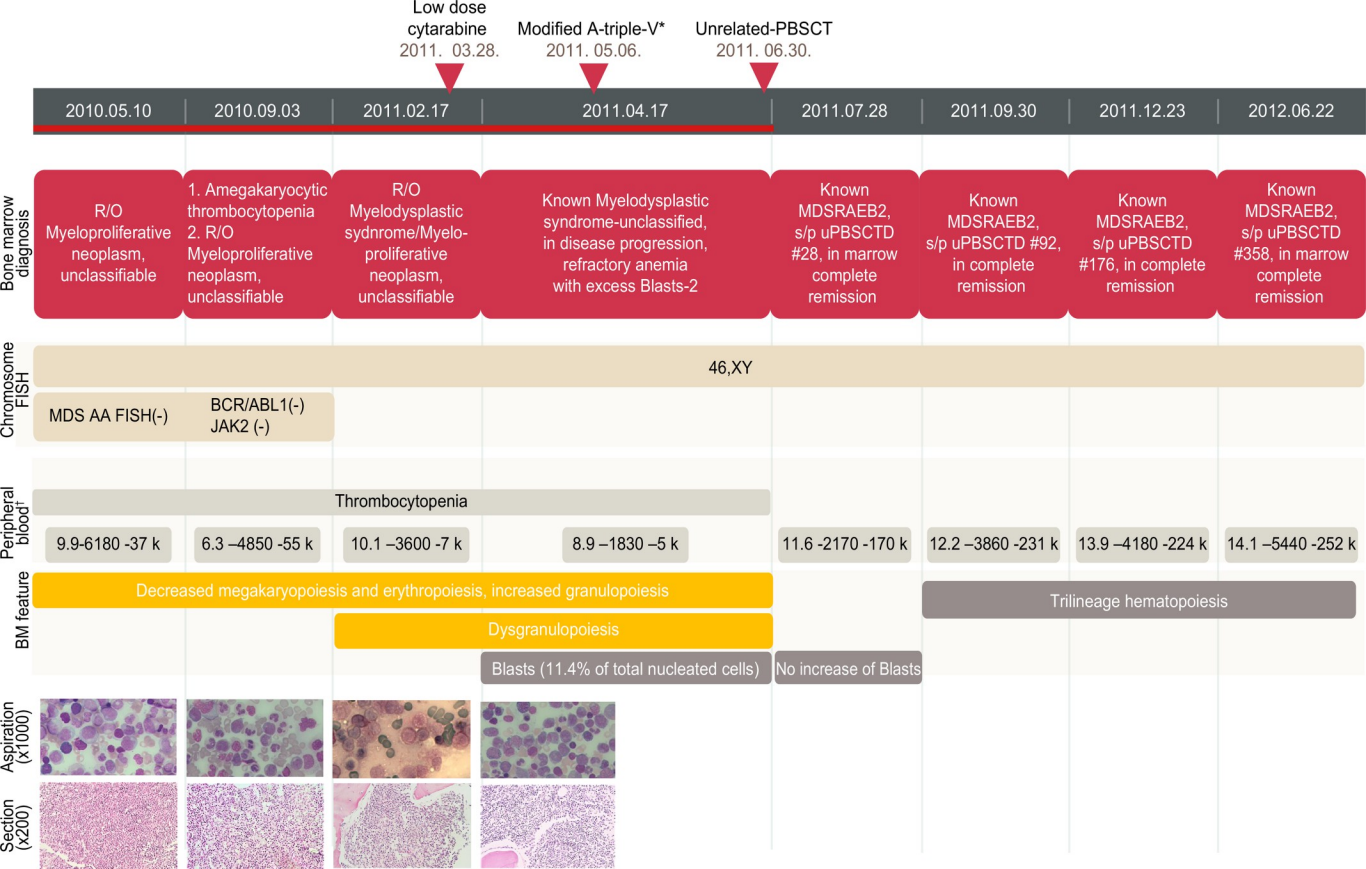
Clinical course of the patient 1: Eight consecutive BM examinations, cytogenetic studies, hemograms, and treatments. FISH, fluorescent in situ hybridization; BM, bone marrow; uPBSCT, unrelated peripheral blood stem cell transplantation; MDS, myelodysplastic syndrome; MDS-RAEB 2, myelodysplastic syndrome; refractory anemia with excess blasts-2. *Etoposide, cytarabine, vincristine, vinblastine.^†^ Blood cell count shows hemoglobin (×10^3^ /L), white blood cell count (×10^6^/L), and platelet count (×10^6^/L) in that order.

The patient 1 (proband, M/9 years) visited the hospital with the chief complaint of recurrent bleeding tendency. The initial hemogram showed anemia, thrombocytopenia, hemoglobin levels of 8.2 × 10^3^ g/L, a WBC count of 6.5 × 10^9^/L, and a platelet count of 22 × 10^9^/L. The proband had a history of taking anti-epilepsy drugs and showed persistent thrombocytopenia despite the discontinuation of antiepileptic drugs. The patient’s first bone marrow examination revealed decreased levels of megakaryopoiesis and erythropoiesis (Fig 3). The bone marrow was hypercellular (cellularity 100%) with marked granulocytic hyperplasia. The bone marrow aspirate was severely diluted by peripheral blood, and nucleated cells were rarely observed in the bone marrow smear. Dysgranulopoietic features, such as hypogranular neutrophils, were observed in the peripheral blood. Anemia and thrombocytopenia refractory to platelet infusion persisted. Initially, the diagnosis was uncertain because the diagnostic criteria for myeloproliferative neoplasm (MPN) or MDS were not satisfied, and the suspected diagnosis was myelodysplastic–myeloproliferative neoplasm, unclassifiable (MDS/MPN-U). Four months later, the hemogram showed hemoglobin levels of 6.3 × 10^3^ g/L, a WBC count of 4.85 × 10^9^/L, and a platelet count of 55 × 10^9^/L. The second bone marrow examination showed lower levels of megakaryopoiesis than that observed in the previous specimen; however, erythroid hyperplasia and bone marrow hypercellularity persisted. Four months later, thrombocytopenia became severe, with hemoglobin levels of 10.1 × 10^3^ g/L, a WBC count of 3.6 × 10^9^/L, and a platelet count of 7 × 10^9^/L. The third bone marrow examination showed a marked decrease in the levels of megakaryocytes (0-1/×400 HPF), with persistent erythroid hyperplasia and granulocytic hyperplasia. Dysgranulopoietic features such as hypogranulation and hypolobular neutrophils were observed in 80% of granulocytic lineages. Low-dose cytarabine treatment was initiated, and after four weeks, the bone marrow study revealed an increase in the number of myeloblasts (11.5% of total bone marrow nucleated cells). The patient was then diagnosed with RAEB2 (refractory anemia with excess blasts-2). The modified A-triple-V (etoposide, cytarabine, vincristine, vinblastine) regimen was administered, and then unrelated peripheral blood stem cell transplantation (uPBSCT) was performed. Four weeks after uPBSCT, the bone marrow showed trilineage hematopoiesis and a decrease in the number of blasts to less than 2.0% among bone marrow nucleated cells. The patient’s hemogram improved, showing hemoglobin levels of 11.6 × 10^3^ g/L, a WBC count of 2.17 × 10^9^/L, and a platelet count of 170 × 10^9^/L. Bone marrow examinations performed 3 months, 6 months, and 1 year after uPBSCT revealed trilineage hematopoiesis with a normal hemogram.

### Cytogenetic analysis of the proband (patient 1) with the *THPO* variant

G-banding and FISH for -5/5q-, -7/7q-, +8, -20/20q-, and +1/1q+ were performed during the first bone marrow examination of patient 1. G-banding showed a normal karyotype, and FISH showed no chromosomal abnormalities (Table 2).

### Family study of the proband (patient 1) with the *THPO* variant

**Fig 4:**
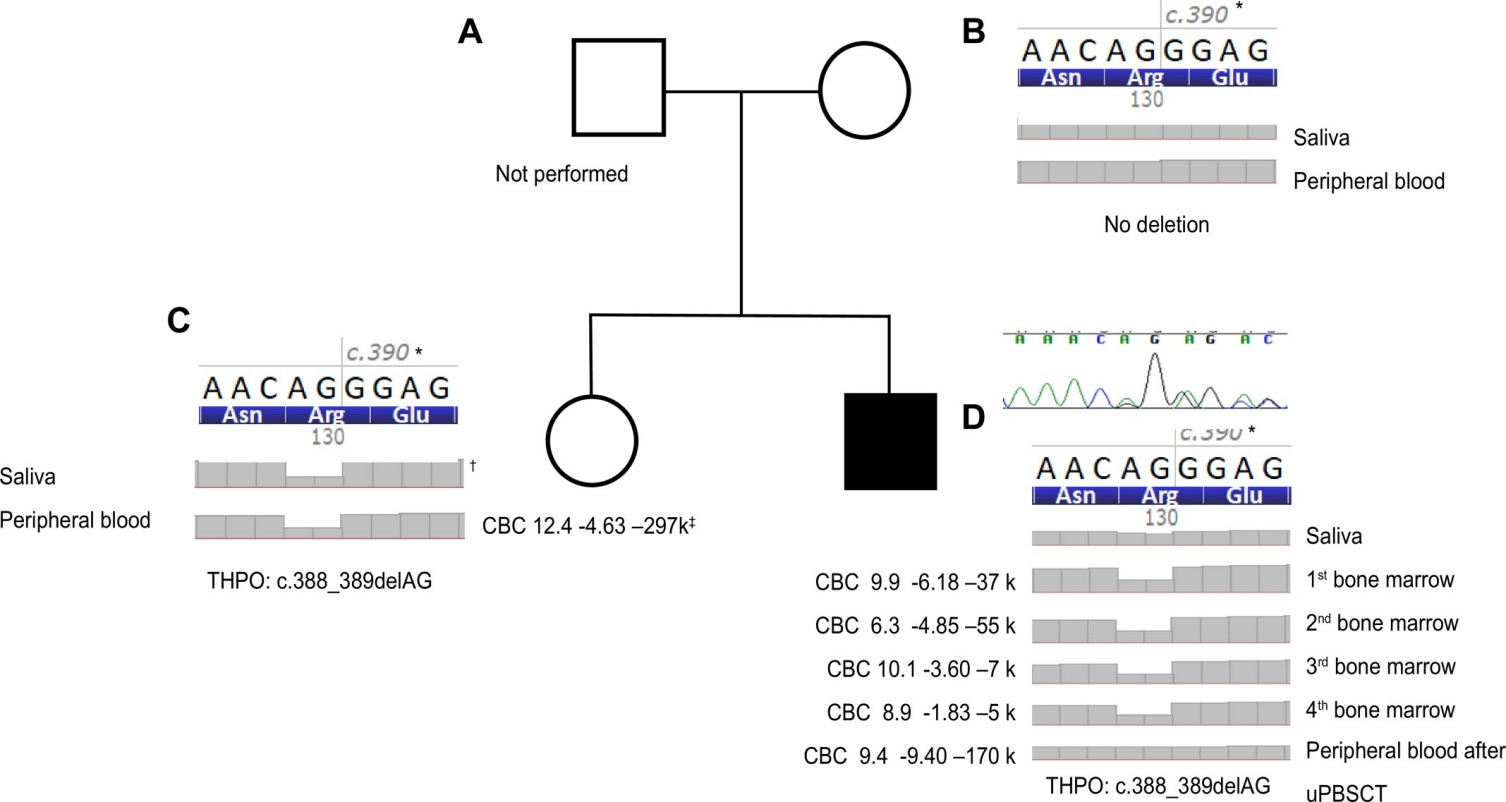
Pedigree of the proband (patient 1) with *THPO* variants and monoallelic deletion of *THPO*: c.388_389delAG. (A). The proband’s father was not included in the family study. (B). No *THPO* deletion was observed in the saliva and peripheral blood samples of the mother. (C). Monoallelic deletion of *THPO*: c.388_389delAG was observed in the saliva and peripheral blood samples of the sister. (D). Monoallelic deletion of *THPO*: c.388_389delAG was observed in four consecutive bone marrow and saliva samples of the proband. However, the deletion was not observed in the peripheral blood sample after uPBSCT. Monoallelic deletion of *THPO*: c.388_389delAG was confirmed by Sanger sequencing in four consecutive bone marrow samples of the proband. CBC: complete blood count; uPBSCT: unrelated peripheral blood stem cell transplantation. *Reference codon and amino acid sequence of NM_001290003.1. are above gray bars. ^†^Gray bars indicate sequencing depth. ^‡^CBC shows hemoglobin (x10^3^/L), white blood cells (x10^9^/L) and platelets (x10^6^/L) in order.

**Fig 5:**
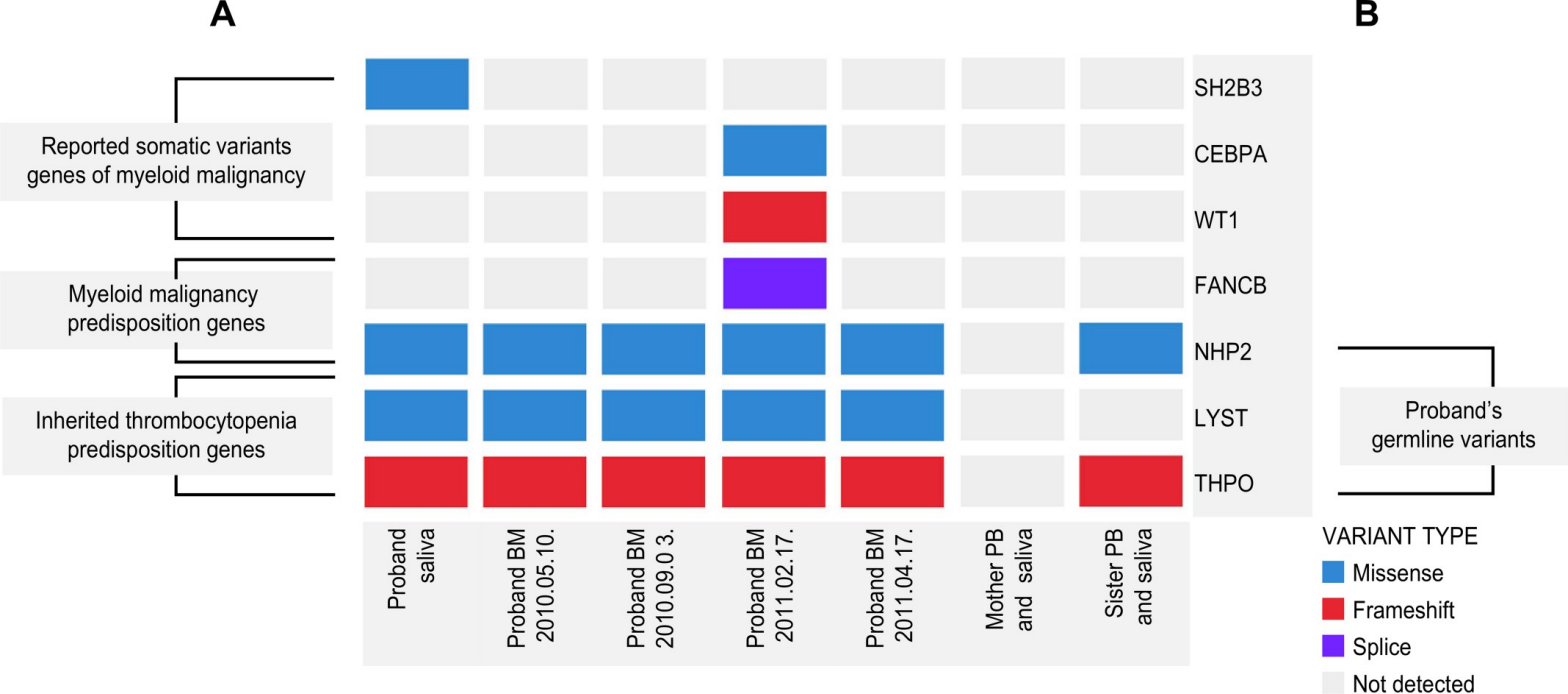
Heatmap showing gene variants in the proband (patient 1) family. (A). Of all variants filtered out by targeted multigene sequencing performed on the proband’s family, only myeloid malignancy predisposition genes, inherited thrombocytopenia predisposition genes, and somatic variants of myeloid malignancy-related genes are represented. Inherited thrombocytopenia predisposition genes were identified based on guidelines in Bury et al. [1]. Myeloid malignancy predisposition genes identified were based on the WHO criteria and NCCN guidelines. The somatic variant was identified based on the NCCN guidelines and reported in the COSMIC database. (B). The variants of *THPO*, *LYST*, and *NHP2* genes were observed in the proband and sister specimens and classified into germline variants of the proband. Because of the absence of the proband’s father’s information, the variants of *FANCB*, *WT1*, *CEBPA*, and *SH2B3* could not be classified as somatic or germline variants. BM, bone marrow; PB, peripheral blood; WHO criteria, World Health Organization classification of tumors of hematopoietic and lymphoid tissues; NCCN, The National Comprehensive Cancer Network; COSMIC, Catalog Of Somatic Mutations in Cancer.

Subsequent targeted multigene sequencing of the same genes as those sequenced in the three MDS patients, including 647 genes, was performed on four consecutive bone marrow specimens, peripheral blood and saliva specimens of the proband, and peripheral blood and saliva specimens of the proband’s mother and sister. The pedigree is shown in Fig 4. The family study was conducted without a father. The filtered variants from the family study are shown in a heatmap (Fig 5). The filtered variant genes were classified as those showing a predisposition to inherited thrombocytopenia, myeloid malignancy, and reported somatic variant genes of myeloid malignancy (Fig 5A). The reported somatic variant genes of myeloid malignancies were in accordance with the NCCN (The National Comprehensive Cancer Network) guidelines and COSMIC (Catalog Of Somatic Mutations In Cancer) databases. The proband’s peripheral blood sample was excluded for the interpretation of germline variants because it was obtained after an unrelated peripheral blood stem cell transplantation. Variants observed in both saliva and bone marrow samples were considered germline variants. The variants filtered from the proband’s four consecutive bone marrow samples that were not detected in the proband’s saliva sample and the duplicate variants filtered from the mother’s and sister’s saliva and peripheral blood samples were also classified as germline variants. The variants filtered only from the proband’s four consecutive bone marrow samples were classified as somatic variants. *THPO*:c.388_389delAG (NM_001290003) was identified in the proband’s saliva sample, four consecutive bone marrow samples, and in the sister’s saliva and peripheral blood samples and was considered a germline variant *THPO*:c.388_389delAG (NM_001290003) was classified as a pathogenic variant, and the other germline variants were classified as VUS. All somatic variants were classified as Tier 3 variants.

### A deleterious monoallelic small deletion variant in *THPO* in the proband and his sister

The family study revealed *THPO*: c.388_389delAG in the proband and his sister but not in the mother. The *THPO*: c.388_389delAG, p.Arg130Gly variant can cause a frameshift change in the 130^th^ amino acid arginine to glycine. This frameshift variant was confirmed by IGV and Alamut Visual 2.15 as a monoallelic deletion (Fig 4B–4E). The same monoallelic deletion of *THPO* was detected in the saliva and peripheral blood of the proband’s sister and in the saliva and in four consecutive bone marrow samples in the proband. After uPBSCT, the peripheral blood of the proband showed no deletion of *THPO*: c.388_389delAG.

To validate the *THPO* variant identified by targeted multigene sequencing, Sanger sequencing was performed on four consecutive bone marrow samples from the proband, and the *THPO*:c. 388_389delAG was confirmed in all samples (Fig 4D).

## Discussion

In this study, we discovered a *THPO* germline variant (*THPO*:c.388_389delAG) in three patients with RCC. Through a family study, we found that the proband’s sister carried the *THPO* variant, while the mother did not. In 2016, the WHO criteria denoted ‘myeloid neoplasms with germline predisposition and preexisting platelet disorder’ [12]. Genes involved in germline predisposition and preexisting platelet disorders include *RUX1*, *ANKRD26*, and *ETV6*. In addition to these three genes, we suggest *THPO* as a potential gene underlying predisposition to myeloid malignancy.

**Fig 6:**
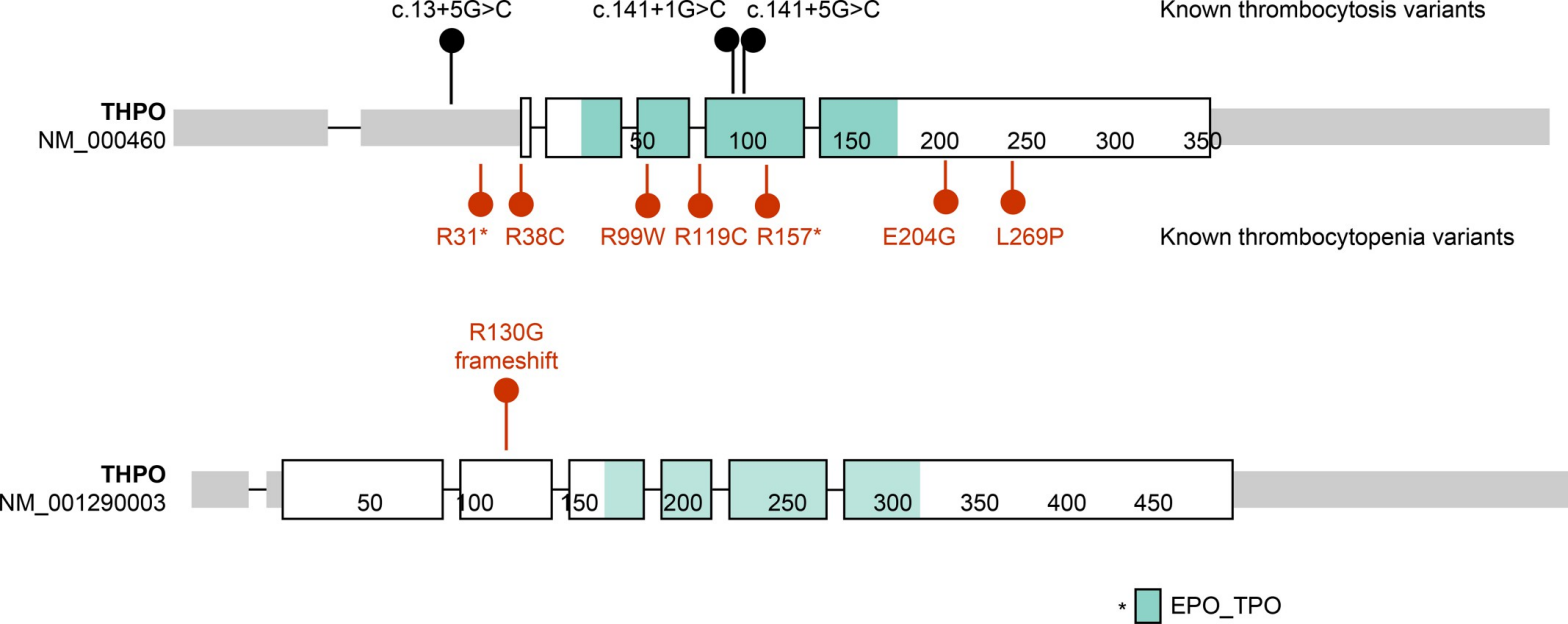
Reference *THPO* gene sequence of NM_000460 and NM_001290003. Known variants of the *THPO* gene sorted by variant type at NM_000460. *THPO* variants found in hereditary thrombocytopenia and thrombocytosis are indicated in red dots and black points, respectively. The reference sequence NM_001290003 contains *THPO*:c.388_389delAG, p. R130G. Image created using ProteinPaint, which is the protein-based visualization tool created at St. Jude.*Green area indicates the coding region of erythropoietin/thrombopoietin in each lollipop.

Previously, variants in the 5′ UTR of *THPO* have been reported as causative variants associated with thrombocytosis, whereas variants in its receptor-binding domain are associated with thrombocytopenia [18–20]. Formerly, *THPO*:c.388_389delAG belonged to the 5’ UTR in the reference sequence (NM_000046.4); however, it has recently been relocated in the coding region of the receptor-binding domain in the reference sequence (NM_001290003.1) (Fig 6). Thus, variants in the formerly classified 5′ UTR of *THPO* can cause inherited thrombocytopenia, as proven by a functional study [21].

The *THPO* gene encoding thrombopoietin can directly affect the first step of megakaryocytic differentiation at the stem cell level. Moreover, recent studies have reported an alternative pathway in which long-term hematopoietic stem cells (LT-HSCs) directly differentiate into megakaryocytes without going through the process of becoming multipotent progenitor cells (MPPs), common myeloid progenitor cells (CMPs), and megakaryocyte–erythroid progenitor cells (MEPs) [22]. Nakamura-Ishizu et al. [23] showed that thrombopoietin regulates hematopoietic stem cell quiescence and mobilization by stimulating hematopoietic stem cell (HSC) entry into the cell cycle. Depending on the location of the variant in *THPO*, patients with *THPO* variants show variable clinical symptoms, ranging from thrombocytosis to thrombocytopenia (Fig 6). In this study, the proband’s sister did not show any thrombocytopenia-related symptoms even in adulthood, which is consistent with the fact that monoallelic *THPO* variants show no or mild symptoms. The coexisting anemia of the proband indicates that *THPO* can induce a wide variety of symptoms during the first stage of hematopoiesis. We could not perform a platelet function study, as the diagnosis of the proband was uncertain at the initial bone marrow study before genetic analysis.

*THPO*-related thrombocytopenia has a different phenotype from that of *MPL*-related CAMT. Monoallelic variants of *THPO* cause thrombocytopenia with mild symptoms [2, 3]. In contrast, biallelic loss-of-function variants cause severe congenital thrombocytopenia, leading to bone marrow failure in both adults and infants; these patients do not respond to hematopoietic stem cell transplantation, unlike those with *MPL*-related CAMT [2, 3, 20]. In this study, the proband received unrelated hematopoietic stem cell transplantation and had not developed thrombocytopenia for ten years.

Genetic alterations affecting the process of megakaryopoiesis, such as *MYH-9*, *ETV6*, *RUNX1*, *and ANKRD26* variants, have been reported to cause leukemic progression and are especially related to the genetic predisposition of childhood leukemia [24]. However, the *THPO* variant identified in this study has not been shown to underlie the predisposition to myeloid malignancy yet. In this study, the proband had prominent thrombocytopenia and anemia during initial hospitalization and was diagnosed with RCC after a year. We suspected RCC of CAMT in the initial bone marrow study. However, high bone marrow cellularity and diluted bone marrow smears, which obscure dysgranulopoietic features, hindered diagnosis. Aplastic anemia and inherited bone marrow failure syndrome were excluded because the bone marrow was hypercellular. Usually, CAMT shows bone marrow failure features [1, 5], but the proband’s bone marrow showed persistent hypercellular features, which affected the timely diagnosis. After a year, the proband had a progression to MDS-EB2. This result suggests that the monoallelic variant of *THPO* causes thrombocytopenia, as previously reported [2, 3, 18–20], and can act as a gene underlying the predisposition to myeloid malignancy.

Before the treatment started, we did not recognize the *THPO* variant of the proband; however, the proband fortunately received unrelated peripheral blood stem cell transplantation, which was not from his sister who harbored the *THPO* variant. The same monoallelic variant of *THPO* was observed in the proband and sister but not in the mother. Although we could not perform genetic analysis in the proband’s father, we inferred that the variant was inherited from his father. Patients with an underlying familial myelodysplastic syndrome/acute myeloid leukemia (MDS/AML) predisposition present with hypocellular MDS at a young age. Although patients with genetic predisposition syndromes have been shown to be initially responsive to immunosuppressive therapy, they ultimately do not achieve long-term remission with immunosuppression [9, 25–28]. Therefore, patients with known genetic predisposition syndromes should be given timely medical attention. In contrast to sporadic or primary MDS, the MDS treatment plan is not guided by prognostic risk stratification, as currently used MDS prognostic models have excluded patients with known genetic predisposition syndromes. In addition, the risk of disease progression can be underestimated [26, 29–31].

Despite the absence of the proband’s father’s information, the family study revealed that the proband’s sister had the same variant, but she did not have any symptoms of thrombocytopenia or leukemic transformation, unlike the proband. This result indicates that the pattern of penetration can differ according to epigenetic differences, even in individuals carrying the same variant. Gene expression is modulated through methods of epigenetic regulation, such as promoter methylation, small interfering RNA, and histone acetylation [32]. The measurement of serum thrombopoietin (TPO) levels, the most direct result of *THPO* gene expression, may help assess the epigenetic status and the *THPO*-MPL axis [20, 22, 33]. The absence of the measurement of serum TPO levels in siblings was a limitation of this study. However, the result of multitarget next-generation sequencing of 647 genes showed considerable differences in the filtered variants between the siblings, and it can be considered that they have significantly different epigenetic modifications. We also conducted a series of multigene target sequencing on the proband’s consecutive bone marrow samples and observed subsequent disease progression. This process clearly showed proband germline variants and sequential changes in the somatic variants. Furthermore, the family study was conducted using the same gene panel test, which allowed clear discrimination between germline variants and somatic variants of the proband. The family study also revealed a germline predisposition of *THPO* alteration in this family. By sequential sampling and family study of multigene target sequencing, the current study strongly suggests that the identified variant of the *THPO* gene manifested as thrombocytopenia and subsequently led to leukemic progression.

Since neither clinical nor laboratory features are pathognomonic for specific genes for inherited thrombocytopenia, genotyping could be very helpful for diagnosis and patient monitoring. Guidelines recommend genotyping for specific gene variants, namely, *ANKRD26*, *ETV6*, *FPD/AML*, *GPS*, *CAMT*, *THPO*, *FLNA*, *MPIG6B*, *PT-VWD*, and *TRPM6*, related to thrombocytopenia [1]. Clinical NGS is important for patient management and treatment decisions. Bone marrow examination, G-banding, and FISH were performed several times on the patient, but these tests were insufficient for an accurate diagnosis. *THPO*-related thrombocytopenia in the proband was revealed using multitarget panel NGS sequencing. Furthermore, gene testing can have a significant effect on the treatment. In particular, when the same inheritance pattern is present in siblings, it can be a critical factor in related hematopoietic stem cell transplantation.

With advances in gene sequencing, our understanding of hereditary thrombocytopenia has improved rapidly, and this knowledge is bridging the gaps in therapeutic needs. Since genes conferring genetic predisposition to pediatric hematologic malignancies were added to the WHO criteria in 2016, the clinical use of panel-based multitarget NGS has increased. Because the family members of pediatric or even adult MDS/AML patients have an increased risk of bone marrow malignancy, the evaluation of germline variants through family studies has become very important in the treatment of patients and risk assessment of families. Thus, if any family member has a history of inherited platelet disorders, additional genetic counseling should be necessary for all family members.

In summary, we identified *THPO* variants in three patients with MDS. By retrospective targeted multigene sequencing, a germline *THPO* variant with microdeletion was identified in one RCC patient (33%, 1/3 MDS). Furthermore, the family study revealed that the same *THPO* variant was present in the proband’s sister but not his mother. Therefore, the siblings shared the same monoallelic *THPO* variant but showed different penetration patterns. A review of the medical history revealed that the patient had inherited thrombocytopenia with a *THPO* variant and showed megakaryocytic hypoplasia. During the initial workup, the diagnosis was uncertain because the patient showed markedly hypercellular marrow with packed bone marrow, which is unusual in inherited thrombocytopenia. Subsequent developments in MDS suggest that germline *THPO* mutations are candidate factors underlying germline predisposition to myeloid malignancy. In addition, this patient with the *THPO* variant calls our attention, as the patient showed atypical bone marrow histology from classic inherited thrombocytopenia. Moreover, multigene-targeting NGS in the proband with a monoallelic deletion in the *THPO* gene was critical for diagnosis. Genetic counseling of family members is important for the prevention, treatment and education of families.

## Supporting information

S1 Table Gene list for 647 gene panel. (DOCX)
