# Supplementary material for: Novel THPO variant in hereditary thrombocytopenia: A potential candidate variant for predisposition to myeloid neoplasm
Source: PLoS One. 2022 Dec 19;17(12):e0271624. doi: 10.1371/journal.pone.0271624 (PMC9762605; doi:10.1371/journal.pone.0271624)
Supplement: S1 Table — (DOCX) [file pone.0271624.s001.docx]

**Supporting Information**

**Table S1. Gene list for 647 gene panel**

| *Hereditary platelet disorder related genes* | | | | | | | | |
| --- | --- | --- | --- | --- | --- | --- | --- | --- |
| ACTB | ANKRD26 | AP3B1 | BLOC1S6 | ETV6 | FYB | GATA1 | GNAQ | GNAS |
| LYST | MPL | ORAI1 | RUNX1 | STIM1 | THPO | WAS |  |  |
|  | | | | | | | | |
| *Myeloid malignancy predisposition genes* | | | | | | | | |
| ACD | ANKRD26 | BLM | BRCA1 | BRCA2 | BRIP1 | CBL | CEBPA | CSF3R |
| CTC1 | DDX41 | DKC1 | ELANE | EPCAM | ERCC4 | ETV6 | FANCA | FANCB |
| FANCC | FANCD2 | FANCE | FANCF | FANCG | FANCI | FANCL | FANCM | G6PC3 |
| GATA1 | GATA2 | GFI1 | HAX1 | IKZF1 | KRAS | MLH1 | MSH2 | MSH6 |
| NAF1 | NBN | NF1 | NHP2 | NOP10 | PALB2 | PAX5 | PMS2 | POT1 |
| PTPN11 | RAD51 | RAD51C | RPL11 | RPL27 | RPL35A | RPL5 | RPS10 | RPS17 |
| RPS19 | RPS24 | RPS26 | RPS27 | RPS7 | RTEL1 | RUNX1 | SAMD9 | SAMD9L |
| SBDS | SLX4 | SRP72 | TERC | TERT | TINF2 | TP53 | TPP1 | WAS |
| WRAP53 | XRCC2 |  |  |  |  |  |  |  |
|  | | | | | | | | |
| *Somatic variants related myeloid malignancy genes* | | | | | | | | |
| ASXL1 | BCOR | CALR | CBL | DDX41 | DNMT3A | ETV6 | EZH2 | FLT3 |
| GATA2 | IDH1 | IDH2 | JAK2 | MPL | NF1 | NPM1 | NRAS | PHF6 |
| RUNX1 | SETBP1 | SF3B1 | SRSF2 | STAG2 | STAT3 | TET2 | TP53 | U2AF1 |
| WT1 | ZRSR2 |  |  |  |  |  |  |  |
|  | | | | | | | | |
| *Other hematologic disorders and cancer related genes* | | | | | | | | |
| ABCA7 | ABCB7 | ABCC1 | ABL1 | ABRAXAS1 | ACKR1 | ACVR2B | ACYP2 | ADA |
| ADRBK1 | AK2 | AKAP13 | AKAP9 | AKT1 | AKTIP | ALK | ALMS1 | ALPK2 |
| AMER1 | ANKRD24 | APC | ARHGAP26 | ARID1A | ARID1B | ARID2 | ARID3A | ARID4B |
| ASXL1 | ASXL2 | ASXL3 | ATM | ATR | ATRX | B2M | BAP1 | BARD1 |
| BAX | BCAS3 | BCL10 | BCL11B | BCL2 | BCL2L1 | BCL2L11 | BCL6 | BCL7A |
| BCL9 | BCOR | BCORL1 | BCR | BICD1 | BIRC3 | BLNK | BMPR1A | BRAF |
| BRCC3 | BRD2 | BRD4 | BRD7 | BRINP3 | BRPF1 | BTG1 | BTG2 | BTG3 |
| BTK | BTLA | BUB1 | BUB1B | CACNA1E | CALR | CARD11 | CARD6 | CASP10 |
| CASP8 | CBL | CBLB | CBLC | CBX5 | CBX7 | CCDC80 | CCND1 | CCND3 |
| CD200 | CD27 | CD36 | CD3D | CD3E | CD40LG | CD58 | CD70 | CD79A |
| CD79B | CDC73 | CDH1 | CDH23 | CDK12 | CDK4 | CDKN1B | CDKN1C | CDKN2A |
| CDKN2B | CDKN3 | CEBPA | CELSR2 | CEP164 | CHD1 | CHD2 | CHD8 | CHEK2 |
| CHRNA3 | CHRNA5 | CHRNB4 | CIAO1 | CIAO2B | CIAO3 | CIC | CIITA | CLPTM1L |
| CMYA5 | CNOT1 | COIL | COL4A2 | COL6A3 | CPNE3 | CREBBP | CRLF2 | CSF1R |
| CSF3R | CSTF2T | CTCF | CTNNB1 | CTSS | CUL9 | CUX1 | CXCL12 | CXCR4 |
| CYLD | DAP3 | DAXX | DCAF4 | DCLK1 | DCLRE1B | DCLRE1C | DDB2 | DDX1 |
| DDX11 | DDX23 | DDX3X | DDX54 | DHX29 | DHX32 | DHX58 | DICER1 | DIS3 |
| DIS3L2 | DKK2 | DLEU1 | DLEU2 | DMD | DNAH9 | DNM2 | DNMT1 | DNMT3A |
| DNMT3B | DST | DYRK4 | EBF1 | ECT2L | EED | EEF1E1 | EGFR | EGR2 |
| EP300 | EPHA2 | EPHA3 | EPHA7 | ERBB2 | ERCC2 | ERCC3 | ERCC5 | ERCC6 |
| ETS1 | ETV3 | ETV6 | EXT1 | EXT2 | FAH | FAM46C | FAS | FASLG |
| FAT4 | FBXO11 | FBXW7 | FGFR2 | FGFR3 | FH | FLCN | FLG | FLT3 |
| FOXL2 | FOXN1 | FOXO1 | FOXP3 | FUBP1 | G6PD | GAR1 | GATA2 | GATA3 |
| GLI1 | GNA11 | GNA13 | GNB1 | GPC3 | GPC4 | GSTP1 | HCK | HDAC2 |
| HDAC3 | HDAC7 | HEATR1 | HIST1H1E | HMBOX1 | HNF1A | HNRNPK | HOXB13 | HRAS |
| HUWE1 | IKZF2 | IKZF3 | IL2RG | IL7R | IRAK1 | IRAK4 | IRF1 | IRF4 |
| IRF8 | ITK | ITPKB | JAK1 | JAK2 | JAK3 | JMJD1C | KDM2B | KDM3B |
| KDM4C | KDM5C | KDM6A | KDM6B | KDR | KIAA0355 | KIF20B | KIT | KLF6 |
| KLHL6 | KLK3 | KMT2A | KMT2B | KMT2C | KMT2D | KRAS | L2HGDH | LAMB4 |
| LAMTOR2 | LEF1 | LIG4 | LMO2 | LRP1B | LRRK1 | LSP1 | LUC7L2 | MAD1L1 |
| MAGT1 | MALT1 | MAP2K1 | MAP2K2 | MAP2K4 | MAP3K1 | MAP3K14 | MAP4K1 | MAPK1 |
| MAX | MDM2 | MED12 | MEF2B | MEF2C | MEN1 | MET | METTL3 | MIR155 |
| MKI67 | MMS19 | MPDZ | MPHOSPH6 | MRE11 | MSMB | MST1R | MTA2 | MTOR |
| MUC16 | MUM1 | MUTYH | MXRA5 | MYB | MYC | MYCN | MYD88 | MYLK2 |
| MYO3A | NAT1 | NAT2 | NCF4 | NF2 | NFE2L2 | NFKB2 | NFKBIA | NFKBIE |
| NHEJ1 | NKX2-1 | NOTCH1 | NOTCH2 | NOTCH3 | NPM1 | NR3C1 | NRAS | NRK |
| NSD1 | NTRK1 | NUP214 | OBSCN | OR6K3 | P2RY8 | PALLD | PAPD5 | PARD3 |
| PARP1 | PARP2 | PASD1 | PASK | PBRM1 | PCLO | PDGFC | PDGFRA | PDGFRB |
| PDS5B | PDSS2 | PHLPP1 | PHOX2B | PIF1 | PIGA | PIGT | PIK3C3 | PIK3CA |
| PIK3R1 | PIM1 | PINX1 | PKD1L2 | PLCG2 | PLEKHG5 | PLRG1 | PML | PMS1 |
| PNP | POLG | POLH | POLR2A | POSTN | POU2F2 | PPP2R1A | PRDM1 | PRDM16 |
| PRDM9 | PRF1 | PRKAR1A | PRKCG | PRKD3 | PRKDC | PRPF3 | PRPF40B | PRPF8 |
| PRSS1 | PTCH1 | PTEN | PTPN11 | PTPN14 | PTPRC | PTPRT | RAB27A | RAC2 |
| RAD21 | RAD50 | RAD51AP1 | RAD51B | RAD51D | RAD54L | RAF1 | RAG1 | RAG2 |
| RAP1A | RAPGEF1 | RARA | RB1 | RBBP4 | RBMX | RECQL | RECQL4 | RECQL5 |
| REL | RELN | RET | RFTN1 | RFX7 | RFXAP | RIPK1 | RIT1 | RMRP |
| RNF213 | RPL19 | RPN1 | RPS14 | RPS6KA6 | RUNX1 | SAMHD1 | SAP130 | SCML2 |
| SCRIB | SDHA | SDHAF2 | SDHB | SDHC | SDHD | SDS | SENP6 | SETBP1 |
| SETD2 | SF1 | SF3A1 | SGK1 | SH2B3 | SH2D1A | SHOC2 | SLC37A4 | SLC7A7 |
| SLITRK6 | SMAD1 | SMAD4 | SMAD7 | SMARCA2 | SMARCA4 | SMARCB1 | SMARCD2 | SMARCE1 |
| SMC1A | SMC3 | SMC5 | SMG1 | SMO | SNRNP200 | SOCS1 | SOS1 | SPEN |
| SPINK1 | SRI | SRRM2 | SRSF2 | SRSF6 | SRSF8 | STAT5B | STAT6 | STK11 |
| STK32A | STK33 | STK36 | STK4 | STN1 | STRIP2 | STX11 | STXBP2 | SUDS3 |
| SUFU | SUMO2 | SUPT5H | SUZ12 | SYK | SYNE1 | TAF1 | TAL1 | TAZ |
| TBL1XR1 | TBX1 | TCF12 | TCF4 | TEN1 | TEP1 | TERF1 | TERF2 | TERF2IP |
| TET1 | TET2 | TGFBR2 | TGM6 | TGM7 | THRB | TMEM127 | TMEM30A | TNFAIP3 |
| TNFRSF14 | TNFSF9 | TNKS | TNKS1BP1 | TNKS2 | TOX3 | TRA2B | TRAF3 | TRIM37 |
| TRIO | TSC1 | TSC2 | TSHR | TTBK1 | TTC27 | TTN | TYK2 | TYW1 |
| U2AF1 | U2AF1L4 | U2AF2 | UBA3 | UBE2A | UGGT1 | ULK4 | UNC13B | UNC13D |
| UNC5C | UNC5D | USB1 | VHL | VPS13A | VPS13B | VPS45 | WAC | WAPAL |
| WEE1 | WIPF1 | WNK3 | WNK4 | WRN | XIAP | XPA | XPC | XPO1 |
| XRCC6 | ZAP70 | ZBTB33 | ZBTB7B | ZEB2 | ZFHX3 | ZMYM3 | ZNF208 | ZNF311 |
| ZNF608 | ZNF676 | ZNF708 | ZRSR2 |  |  |  |  |  |
|  | | | | | | | | |
